# Tripartite motif 38 alleviates the pathological process of NAFLD–NASH by promoting TAB2 degradation

**DOI:** 10.1016/j.jlr.2023.100382

**Published:** 2023-04-26

**Authors:** Xinxin Yao, Ruixiang Dong, Sha Hu, Zhen Liu, Jie Cui, Fengjiao Hu, Xu Cheng, Xiaoming Wang, Tengfei Ma, Song Tian, Xiao-Jing Zhang, Yufeng Hu, Lan Bai, Hongliang Li, Peng Zhang

**Affiliations:** 1 Taikang Medical School (School of Basic Medical Sciences), Wuhan University, Wuhan, China; 2 Institute of Model Animal, Wuhan University, Wuhan, China; 3 Medical Science Research Center, Zhongnan Hospital of Wuhan University, Wuhan, China; 4 Gannan Innovation and Translational Medicine Research Institute, Ganzhou, China; 5 Key Laboratory of Cardiovascular Disease Prevention and Control, Ministry of Education, First Affiliated Hospital of Gannan Medical University, Gannan Medical University, Ganzhou, China; 6 Department of Neurology, Huanggang Central Hospital, Huanggang, China; 7 Department of Cardiology, Renmin Hospital of Wuhan University, Wuhan, China

**Keywords:** TRIM38, NAFLD, metabolism disorder, hepatic steatosis, TAB2, MAPK

## Abstract

Nonalcoholic fatty liver disease (NAFLD) has become the most prevalent chronic liver disease worldwide, without any Food and Drug Administration-approved pharmacological intervention in clinic. *Trim38*, as an important member of the TRIM (tripartite motif-containing) family, was largely reported to be involved in the regulation of innate immune and inflammatory responses. However, the functional roles of TRIM38 in NAFLD remain largely unknown. Here, the expression of TRIM38 was first detected in liver samples of both NAFLD mice model and patients diagnosed with NAFLD. We found that TRIM38 expression was downregulated in NAFLD liver tissues compared with normal liver tissues. Genetic *Trim38*-KO in vivo showed that TRIM38 depletion deteriorated the high-fat diet and high fat and high cholesterol diet-induced hepatic steatosis and high fat and high cholesterol diet-induced liver inflammation and fibrosis. In particular, we found that the effects of hepatocellular lipid accumulation and inflammation induced by palmitic acid and oleic acid were aggravated by TRIM38 depletion but mitigated by TRIM38 overexpression in vitro. Mechanically, RNA-Seq analysis demonstrated that TRIM38 ameliorated nonalcoholic steatohepatitis progression by attenuating the activation of MAPK signaling pathway. We further found that TRIM38 interacted with transforming growth factor-β-activated kinase 1 binding protein 2 and promoted its protein degradation, thus inhibiting the transforming growth factor-β-activated kinase 1-MAPK signal cascades. In summary, our study revealed that TRIM38 could suppress hepatic steatosis, inflammatory, and fibrosis in NAFLD via promoting transforming growth factor-β-activated kinase 1 binding protein 2 degradation. TRIM38 could be a potential target for NAFLD treatment.

Nonalcoholic fatty liver disease (NAFLD) has become a predominant cause of chronic liver dysfunction with a worldwide prevalence of more than 25% (1, 2). NAFLD constitutes a broad spectrum of liver dysfunction; among them, nonalcoholic steatohepatitis (NASH) is the advanced stage of NAFLD, and often accompanied by insulin resistance and steatosis, as well as evidence of hepatocyte injury and inflammation infiltration either with or without fibrosis (3). Patients with NASH have an increased risk of progression to advanced fibrosis, cirrhosis, and even hepatocellular carcinoma. Although many clinical and experimental studies have been performed, the detailed molecular mechanisms involved in the pathogenesis of NASH remain to be elucidated (4). Unfortunately, there is no Food and Drug Administration-approved pharmacotherapy for NASH yet (5, 6). Thus, there is an unmet need to uncover therapeutic targets and explore feasible strategies for NASH treatment.

The superfamily of tripartite motif-containing (TRIM) proteins is the largest really interesting-new-gene domain-containing E3 ligases that exert multiple functions in antiviral responses, innate immunity, and inflammatory responses (7, 8). Similar to the other TRIM family members, TRIM38 has been first reported to be a critical effector in the generation and development of viral infection and inflammatory diseases (9). TRIM38 serves as a negative regulator for Toll-like receptor and retinoic acid-inducible gene-I-mediated IFN-β production in host antiviral response (10). TRIM38 also protects chondrocytes from IL-1β-induced apoptosis and degeneration by suppressing NF-κB pathway (11). In H9C2 cells, TRIM38 overexpression relieves inflammatory responses, oxidative stress, and apoptosis in myocardial ischemia/reperfusion injury by inhibiting transforming growth factor beta-activated kinase 1 (TAK1)/NF-κB pathway (12). TRIM38 is thought to have E3 ubiquitin ligase activity. In mouse RAW264.7 cells, TRIM38 inhibits Toll-like receptor 3/4-mediated NF-κB activation by targeting TRAF6 for ubiquitination and degradation (13). Furthermore, TRIM38 also interacts with NAP1, triggering ubiquitination and proteasomal degradation in the antiviral immune response (10). Although the major function of TRIM38 depends on its E3 ligase activity, we notice that TRIM38 also regulates the protein stability independent of its ubiquitin ligase activity. Hu *et al.* (14) showed that TRIM38 targeted STING for sumoylation and thus promoted STING activation and protein stability during the early phase of viral infection. TRIM38 negatively regulates proinflammatory signaling, MAPK pathway, and NF-κB activation by mediating lysosome-dependent degradation of TAK1-binding protein 2/3 (TAB2/3) (15, 16, 17). These findings indicate that TRIM38 exhibits specific function and mechanism under the different pathological conditions. Previous reports reveal that TAK1-TAB2/3-MAPK pathway is the essential hub in the initiation and progression of NAFLD, including lipid metabolism, inflammation, and liver injury (18, 19), thus we speculate that TRIM38 might participate in the regulation of NAFLD.

In this study, we observed that TRIM38 was markedly downregulated in NAFLD. We further proved that TRIM38 depletion aggravated the high-fat diet (HFD)- or high fat and high cholesterol (HFHC) diet-induced hepatic steatosis, inflammation, and fibrogenesis in mice. Mechanistically, we found that TRIM38 interacted with TAB2 and promoted its protein degradation, thus attenuating the activation of the MAPK signaling pathway. Our findings reveal the mechanisms of TRIM38 in NAFLD and support a potential that targeting TRIM38-MAPK axis is a novel strategy for NAFLD treatment.

## Materials and methods

### Human liver samples

Human liver tissues were obtained from individuals who underwent liver surgery and transplantation from Zhongnan Hospital of Wuhan University (Wuhan). Informed consents were obtained from the indicated patients and volunteers. This study protocol conformed to the ethical guidelines of the Declaration of Helsinki Principles and was approved by the Ethics Committee of the Zhongnan Hospital of Wuhan University.

### Animal models

All animal protocols were approved by the Animal Care and Use Committee of Renmin Hospital of Wuhan University. About 8–10-week-old male mice of 21–27 g of weight were included in this study. All mice used for experiment were housed in a specific pathogen-free animal laboratory in a temperature- (24 ± 2°C) and humidity-controlled (40–70%) environment with 12 h light/12 h dark cycle. To establish a mouse model of NAFLD–NASH, male C57BL/6J mice were subjected to 24-week HFD (protein, 20%; fat, 60%; carbohydrate, 20%; H10060, Beijing HUAFUKANG Bioscience Co, Ltd) or 16-week HFHC (protein, 14%; fat, 42%; carbohydrate, 44%; cholesterol, 2%; TP26304; Trophic Diets, Nantong) or 4-week methionine-choline-deficient diet (MCD; TP3005G; Trophic Diets) feeding. Mice of control group were fed with normal chow diet (protein, 18%; fat, 4%; carbohydrate, 78%; catalog no.: 1010001, Jiangsuxietong) or methionine-choline sufficient diet (catalog no.: TP3005GS; Trophic Diets). The animals received humane care according to the criteria outlined in the Guide for the Care and Use of Laboratory Animals prepared by the National Academy of Sciences and published by the National Institutes of Health.

### Generation of genetically modified mice

*Trim3*8-KO mice were obtained by Cas9 system. First, the CRISPR online design tool (http://chopchop.cbu.uib.no/) was used to predict target DNA region boot sequence—guideRNA target site: GCAATGTCAGCCCAAAAACAGGG. Then, the expression vector of *Trim38*-sgRNA was constructed through pUC57-sgRNA (Addgene; catalog no.: 51132). Using the newly constructed pUC57-*Trim38*-sgRNA as a template, a DNA fragment containing the T7 promoter and sgRNA was obtained by PCR. The PCR products and a Cas9-expressed plasmid pST1374-Cas9 (Addgene; catalog no.: 44758) were transcribed in vitro with a T7 ULTRA Transcription Kit (catalog no.: AM1345; Invitrogen). Cas9 mRNA and sgRNA were purified with a Transcription Clean-Up Kit (catalog no.: AM1908; Invitrogen). The purified Cas9 mRNA and sgRNA were injected into C57BL/6 mouse single-cell fertilized egg by FemtoJet 5247 microinjection system. The injected fertilized eggs were transplanted into surrogate female mice, and F0 generation mice were obtained after about 19–21 days of pregnancy. Genomic DNA was extracted from the toes of mice 2 weeks after birth. The primers (*Trim38*-check F1: 5′-TGGGCTCAGACTTTAGCACG-3′ and *Trim38*-check R1: 5′-TCTTCCCAATAACAGCGCCA-3′) were used to identify the genotypes of mice.

### Physiological parameter detection

The body weight (BW) and blood glucose of mice were monitored every 4 weeks, and the mice were fasted for 6 h before each monitoring. Then, glucose tolerance test (GTT) assays were performed as described. After fasting for 6 h, the BW and blood glucose content of the mice were measured, followed by intraperitoneal injection of glucose at a dose of 1 g/kg. The blood glucose content of the mice was measured at 15, 30, 60, and 120 min after the injection, and then the area under the curve (AUC) was calculated using the conventional trapezoid rule. At the end of the experiment, liver weights (LWs) were determined and serum samples were obtained, and levels of serum lipid triglycerides (TGs), total cholesterol (TC), and activities of alanine aminotransferase (ALT) and aspartate aminotransferase (AST) were measured by HITACHI 3110 Automatic Analyzer (HITACHI, Japan) according to the manufacturer’s protocol. Adipose mass was determined by weight of epididymal fat tissue, a common method for evaluating white adipose tissue (20).

### Histopathological analysis

Liver sections from formaldehyde-fixed and paraffin-embedded liver tissues of experimental mice in a thickness of 5 μm were used for H&E staining (hematoxylin; G1004, Servicebio Co, Ltd, Wuhan; eosin, BA-4024, Baso Co, Ltd, Zhuhai) to evaluate the morphological change of liver tissue and lipid accumulation. Frozen sections from liver tissues embedded in optimum cutting temperature compound in a thickness of 8 μm were used for Oil Red O staining (catalog no.: O0625; Sigma-Aldrich, St. Louis, MO) for fat content observation. Picrosirius red (catalog no.: 26357-02; Hede Biotechnology Co, Ltd, Beijing) staining was carried out to visualize the degree of liver fibrosis.

The expression of TRIM38 in clinical samples was analyzed by immunohistochemistry. The sections were subjected to high-pressure antigen repair in EDTA (pH = 9.0) tissue antigen repair solution and incubated with primary antibody TRIM38 (1:100 dilution; Proteintech) overnight at 4°C. The sections were washed with PBS after incubation. Then, the Rabbit Two-step Detection Kit (Rabbit Enhanced Polymer Detection System) (ZSGB-BIO, PV-9001, Beijing) was applied according to the instructions. DAB (ZSGB-BIO, ZLI-9018, Beijing) was used for color development, and hematoxylin was used to stain the nucleus. All open field sections were collected under a light microscope (ECLIPSE 80i; Nikon, Tokyo, Japan).

To analyze the number of inflammatory cells in different groups, CD11b expression was detected by immunofluorescence. Liver sections (5 μm) were subjected to high-pressure antigen repair in EDTA (pH = 9.0) tissue antigen repair solution, and primary antibody CD11b (1:800 dilution, BM3925, Boster, Wuhan) was conjured with fluoro group (Alexa Fluor 568 Goat Anti-Rabbit IgG [H + L], A11036, Invitrogen, Carlsbad, CA) overnight and placed at 37°C for 1 h. Fluorescence microscopy (ECLIPSE 80i) was used for visual field acquisition.

### Primary cell isolation and cell culture

Murine primary hepatocytes were isolated from 6- to 8-week-old male mice using two-step collagenase perfusion and low-speed gradient centrifugation methods as previously described (21). In brief, mice were anesthetized and perfused two times with liver perfusion medium (catalog no.: 17701-038; Thermo Fisher Scientific, Waltham, MA) and liver digestion medium (catalog no.: 17701-034; Thermo Fisher Scientific) via the liver portal vein. After digestion with collagenase, the liver tissues were washed and filtered through a 100 μm cell strainer. Hepatocytes were separated by centrifugation at 50 *g*, and the cells were cultured with DMEM containing 10% FBS and 1% penicillin-streptomycin.

293T cells were cultured in DMEM supplemented with 10% FBS (catalog no.: F05-001-B160216; Bio-One Biotechnology, Guangzhou) and 1% penicillin-streptomycin (catalog no.: 15140-122; Gibco by Invitrogen, Carlsbad, CA) in a 5% CO_2_ incubator at 37°C.

### Cellular Nile red staining

Palmitic acid (PA; P0500; Sigma-Aldrich) and oleic acid (OA; O-1008; Sigma-Aldrich) stimulation was used to construct hepatocyte NAFLD model in vitro as previously reported (22). Fatty acid-free BSA (0.5%; BAH66-0100; Equitech Bio, Kerrville, TX) was used as a control. For Nile red staining, cells were fixed with 4% paraformaldehyde and stained with Nile red (catalog no.: 22190; 1 μM in PBS; Fanbo Biochemicals Co, Ltd, Beijing). Images were acquired with a laser scanning confocal microscope (TCS SP8; Leica, Wetzler, Germany).

### Adenovirus construction and transduction

To overexpress TRIM38 in murine primary hepatocytes, we cloned the entire gene coding sequence into the pENTR-CMV-ATG-FLAG-T2A-EGFP vector. The plasmid was recombined with the pAd/PL-DEST vector using Gateway technology according to the manufacturer’s instructions and then transfected into 293A cells. Adenoviruses were generated using the ViraPower Adenoviral Expression System according to the manufacturer’s instructions (V493-20; Invitrogen, CA). Recombinant adenoviruses were plaque-purified by cesium chloride density gradient centrifugation and verified by restriction digestion. Similar adenoviral vector that carries nonfunctional gene (Ad-control) was used as controls. After plaque purification of the recombinant adenoviruses, the recombinant adenoviruses were diluted to a titer of 10^10^ plaque-forming units per milliliter. Primary hepatocytes were infected with adenovirus supernatant at a multiplicity of infection of 200, and the medium was replaced after 24 h of infection. Cells were harvested at 36 h after infection. All procedures involving viruses were performed in a biosafety cabinet. Primers are listed in supplemental Table S3.

### Western blot assay

For Western blot (WB) analysis, total protein was isolated from cultured cells and liver tissue samples using RIPA lysis buffer mixed with protease inhibitor cocktail tablets (Roche; 04693132001). A BCA Protein Assay Kit (Thermo Fisher Scientific; catalog no.: 23225) was used to determine the protein concentration. The protein samples were separated by 10% SDS-PAGE, transferred to PVDF membranes (Millipore; catalog no.: IPVH00010), and blocked with 5% skim milk for 60 min at room temperature. Then, the membranes were incubated with primary antibodies overnight at 4°C, followed by incubation with the corresponding secondary antibodies, and images were obtained using a ChemiDoc MP Imaging System (Bio-Rad, Hercules, CA). Antibodies are listed in supplemental Table S1.

### RNA extraction and real-time PCR

Total RNA from cells and tissues was extracted with TRIzol reagent (catalog no.: T9424; Sigma-Aldrich), and gene expression was quantified on a real-time PCR System (LightCycler 480 Instrument II; Roche Diagnostics, Inc, Basel, BS, Switzerland) according to the manufacturers’ procedures. The relative gene expression levels of the target genes were normalized to those of the housekeeping gene *ACTB*. The primers used for quantitative RT-PCR (qRT-PCR) are listed in supplemental Table S2.

### Immunoprecipitation assays

Immunoprecipitation (IP) assays were performed as described previously (23). Cells were cotransfected with the indicated plasmids and harvested after 24 h. Then, the cells were washed with ice-cold PBS and lysed with IP lysis buffer (20 mM Tris-HCl, pH 7.4; 150 mM NaCl; 1 mM EDTA; and 1% NP-40) containing protease inhibitor cocktail. After centrifugation at 4°C for 10 min, the supernatant protein was immunoprecipitated with the indicated antitag antibodies and protein G agarose beads overnight at 4°C. The immune complexes were washed three times with NaCl buffer and boiled with 2× SDS loading buffer prior to analysis by WB.

### RNA-Seq and data processing

Total RNA was extracted from liver tissues of different groups, and complementary DNA libraries were constructed. The single-end libraries were sequenced using the MGISEQ 2000 platform (MGI Tech Co, Ltd, Shenzhen). HISAT2 software (version 2.21) was used to align clean reads based on Ensemble reference genomes. SAMtools (version 1.4) was used to sort and convert the mapped reads to BAM format. StringTie (version 1.3.3b) was used to identify the fragments per kilobase per million and read counts. Finally, DESeq2 was used to calculate differential gene expression and statistical significance (fold change > 2 and *P* < 0.05).

### Clustering analysis

In order to evaluate the difference of data between groups, the unweighted mean distance algorithm was used to generate cluster trees for hierarchical cluster analysis. The gene expression of each biological replicate was normalized by z-score method.

### Gene set enrichment analysis

The genes involved in each Kyoto Encyclopedia of Genes and Genomes (KEGG) pathway or Gene Ontology biological process term are defined as a gene set. The “Signal2Noise” metric was used to generate a sorted list and “gene set” permutation type to implement gene set enrichment analysis (GSEA) using the Java GSEA (version 3.0) platform as previously reported (24).

### KEGG pathway enrichment analysis

The KEGG pathway information was downloaded from the KEGG database, and the KEGG pathway annotation and enrichment analysis were carried out by using R script. The pathways with *P* < 0.05 were significantly enriched.

### Statistical analysis

All data were analyzed by SPSS software (version 19.0; IBM Corporation, Armonk, NY). For data showing a normal distribution, differences between two groups were evaluated by Student’s *t*-test, whereas one-way ANOVA followed by the Bonferroni post hoc test (for data showing homogeneity of variance) or Tamhane T2 post hoc test (for heteroscedastic data) was applied for comparisons among multiple groups. Groups of non-Gauss distribution were subjected to nonparametric test. The statistical methods used and the corresponding *P* values for the data shown in each figure panel are included in the figure legends. All data were expressed as the mean ± SD. *P* values are shown as ∗*P* < 0.05, ∗∗*P* < 0.01. The *P* value of less than 0.05 was considered statistically significant.

## Results

### The expression of TRIM38 was repressed during the NAFLD process on protein level

**Fig. 1:**
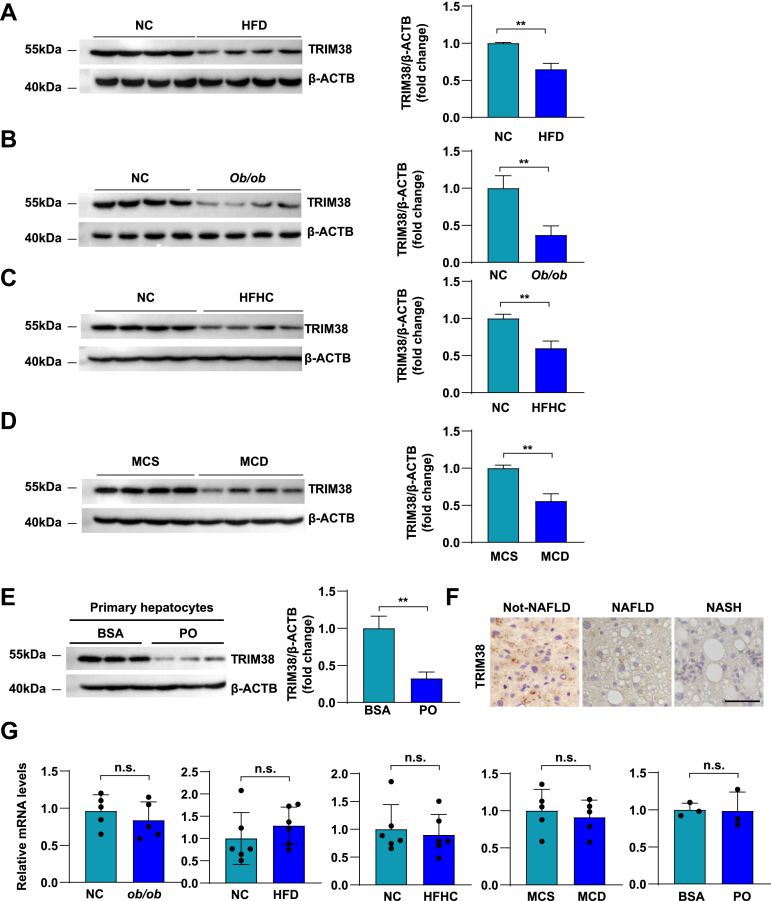
The expression of TRIM38 was repressed during the NAFLD process on protein level. A: Representative WB analysis and quantitative results of TRIM38 protein expression in liver tissue of normal chow (NC) control or HFD-feeding mice. β-actin was used as internal control, *n* = 4 mice/group. B: Representative WB analysis and quantitative results of TRIM38 protein expression in liver tissue of obese (*ob/ob*) mice. β-actin was used as internal control, *n* = 4 mice/group. C: WB assay showed the protein levels of TRIM38 protein expression in liver tissue of NC control or HFHC-feeding mice. β-actin was used as internal control, *n* = 4 mice/group. D: Representative WB analysis and quantitative results of TRIM38 protein expression in liver tissue of NC control or MCD-feeding mice. β-actin was used as internal control, *n* = 4 mice/group. E: Representative WB analysis and quantitative results of TRIM38 protein expression in primary hepatocytes treated with BSA or PO (PA/OA). β-actin was used as internal control, *n* = 3 independent experiments. F: Representative immunohistochemical analysis staining to assess TRIM38 expression in liver sections from human liver sections of NAFL and NASH (*n* = 5 individuals/group). Scale bar represents 50 μm. G: Relative mRNA levels of *Trim38* in liver tissues and primary hepatocytes in the indicated groups. The mRNA expression levels were normalized to those of *ACTB*. For liver tissues of mouse models, *n* = 5–6 mice/group; for primary hepatocytes of corresponding groups, *n* = 3 independent experiments. The data are presented as the mean ± SD values (∗*P* < 0.05, ∗∗*P* < 0.01, n.s., no significance).

To investigate the correlation between TRIM38 and NAFLD, we first determined TRIM38 expression in HFD diet-induced NAFLD mouse models. Compared with normal controls, TRIM38 showed significantly downregulated protein levels in liver tissues of HFD diet-induced NAFLD group detected by WB (Fig. 1A). The same phenomenon was observed in genetic (*ob*/*ob*) obese mice (Fig. 1B). We next examined the expression levels of TRIM38 in the HFHC and MCD diet-induced NAFLD mouse model. Compared with the corresponding controls, the liver tissues showed markedly downregulated protein levels of TRIM38 (Fig. 1C, D). Consistence with the results in mouse models, we further found that TRIM38 expression was specifically downregulated in the hepatocytes upon PA/OA pressure (Fig. 1E). Furthermore, immunohistochemical analysis showed that the expression of TRIM38 was markedly downregulated in liver sections of NAFL and NASH patients (Fig. 1F). To further investigate the regulation of *Trim38* expression in response to metabolic stress, qPCR assay was performed in NAFLD models. Results showed that there was no significant effect on transcriptional level in liver tissues from genetic (*ob/ob*) obese mice and HFD, HFHC, or MCD diet-induced NAFLD mouse models compared with normal controls; the same phenomenon was observed in PA/OA-treated mouse primary hepatocytes (Fig. 1G). Taken together, TRIM38 was significantly downregulated in protein level of liver tissues and primary hepatocytes subjected to metabolic stress rather than in transcriptional level, these findings suggest that TRIM38 might be involved in the pathogenesis of NAFLD via post-transcriptional mechanisms potentially.

### TRIM38 deficiency aggravates HFD-induced insulin resistance and hepatic steatosis

**Fig. 2:**
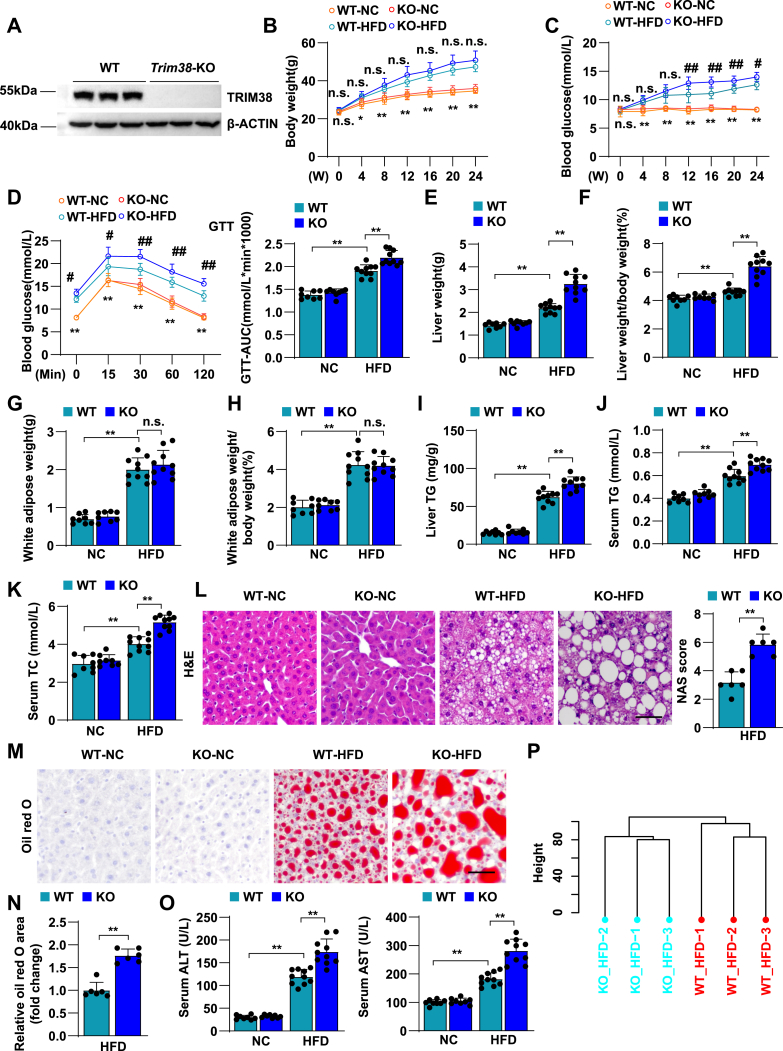

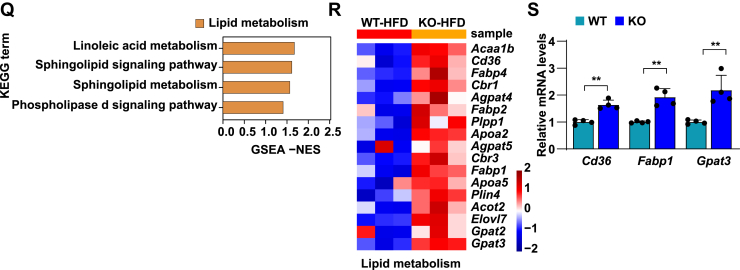
TRIM38 deficiency aggravates HFD-induced hepatic steatosis and insulin resistance. A: Representative WB analysis for *Trim38*-KO detection in liver tissues from both WT and *Trim38*-KO mice under normal chow (NC)-feeding conditions. B, C: BWs (B) and fasting blood glucose concentrations (C) of *Trim38*-KO mice and WT control mice after fed with HFD or NC diet for indicated weeks (*n* = 8–10 mice/group). D: GTT tolerance was tested in *Trim38*-KO mice and WT control mice at the 23rd week of NC or HFD feeding, and the corresponding areas under the curve were calculated (*n* = 8–10 mice/group). E–H: LW (E), LW-to-BW ratio (F), white adipose weight (G), and white adipose-to-BW ratio (H) of *Trim38*-KO mice and WT control mice after NC or HFD treatment for 24 weeks, *n* = 8–10 mice/group. I–K: Liver TG (I), serum TG (J), and serum TC (K) levels were performed in *Trim38*-KO mice and WT control mice after NC or HFD consumption for 24 weeks, *n* = 8–10 mice/group. L–N: Representative images of H&E staining (L) and Oil Red O staining (M) of liver sections of HFD-fed *Trim38*-KO mice and WT control mice. NAS scores and Oil Red O area were quantified respectively. *n* = 6 mice/group. Scale bars represent 50 μm. O: The serum levels of ALT and AST in the indicated mice groups. *n* = 8–10 mice/group. P: Hierarchical clustering showed the distribution and distances of liver samples from *Trim38*-KO mice and WT control mice post 24 weeks of feeding of HFD. Q: GSEA showed the major activated pathways involved in lipid metabolic processes according to the transcriptomics data in liver tissue from HFD-induced *Trim38*-KO mice and WT control mice. R: Heatmap exhibited the leading genes involved in lipid metabolic pathway of HFD-induced KO mice compared with HFD-induced WT mice from RNA-Seq datasets. S: Relative mRNA levels of lipid metabolism genes in the indicated groups. *n* = 4 mice/group. The mRNA expression levels were normalized to those of *ACTB*. The data are presented as the mean ± SD values. B–D: ∗ indicates a statistical difference between the WT-NC group and WT-HFD group. # indicates a statistical difference between the WT-HFD group and the KO-HFD group (∗*P* < 0.05, ∗∗*P* < 0.01, ^#^*P* < 0.05, ^##^*P* < 0.01, n.s., no significance). The data in L and S were statistically analyzed with a two-tailed Student’s test. The data in N were analyzed with nonparametric test, and the data in other panels were analyzed with one-way ANOVA.

Given the fact that the expression of TRIM38 is downregulated in NAFLD, we further generated a *Trim38* systemic KO (*Trim38*-KO) (Fig. 2A) mouse strain via CRISPR-Cas9 system to reveal the function of TRIM38 in the pathology of NAFLD process in vivo. An HFD feeding for 24 weeks was used to establish NAFLD model. WB analysis demonstrated the successful KO of TRIM38 protein in *Trim38*-KO mice (Fig. 2A). After 24 weeks of HFD feeding, we found that mice with TRIM38 ablation showed negligible effect on BW (Fig. 2B). In addition, compared with the control group, TRIM38 deficiency markedly enhanced the HFD-induced glucose metabolic disorder, as evidenced by the higher glucose levels under fasting conditions and increased AUC of GTTs compared with the control group (Fig. 2C, D). Importantly, we found that mice with TRIM38 ablation showed higher LWs and higher LW/BW ratios than WT mice (Fig. 2E, F), whereas TRIM38 showed almost no impact on white adipose (Fig. 2G, H). Notably, the TG or TC concentration in the livers and serum of the *Trim38*-KO mice also increased compared with the control group (Fig. 2I–K). In addition, *Trim38*-KO mice exhibited exacerbated hepatic steatosis, as indicated by H&E and Oil Red O staining (Fig. 2L, M). The elevated AST and ALT levels in serum indicated the occurrence of more severe liver damage in *Trim38*-KO mice than in WT controls (Fig. 2O).

To profile the gene expression differences resulted from TRIM38 deletion during NAFLD, the livers of *Trim38*-KO and WT mice were subjected to RNA-Seq analysis after 24 weeks of HFD feeding. Based on the transcriptomic analysis, hierarchical clustering showed that samples were clearly distributed into WT-HFD and KO-HFD categories (Fig. 2P). Notably, lipid metabolic-related pathways were significantly activated in the KO-HFD group by GSEA, including linoleic acid metabolism, sphingolipid signaling pathway, sphingolipid metabolism, and phospholipase signaling pathway (Fig. 2Q, R). We also confirmed the RNA-Seq data by performing qPCR assay and examining mRNA levels of representative genes (Fig. 2S).

### TRIM38 deficiency aggravates HFHC-induced hepatic steatosis, inflammation, and fibrosis

**Fig. 3:**
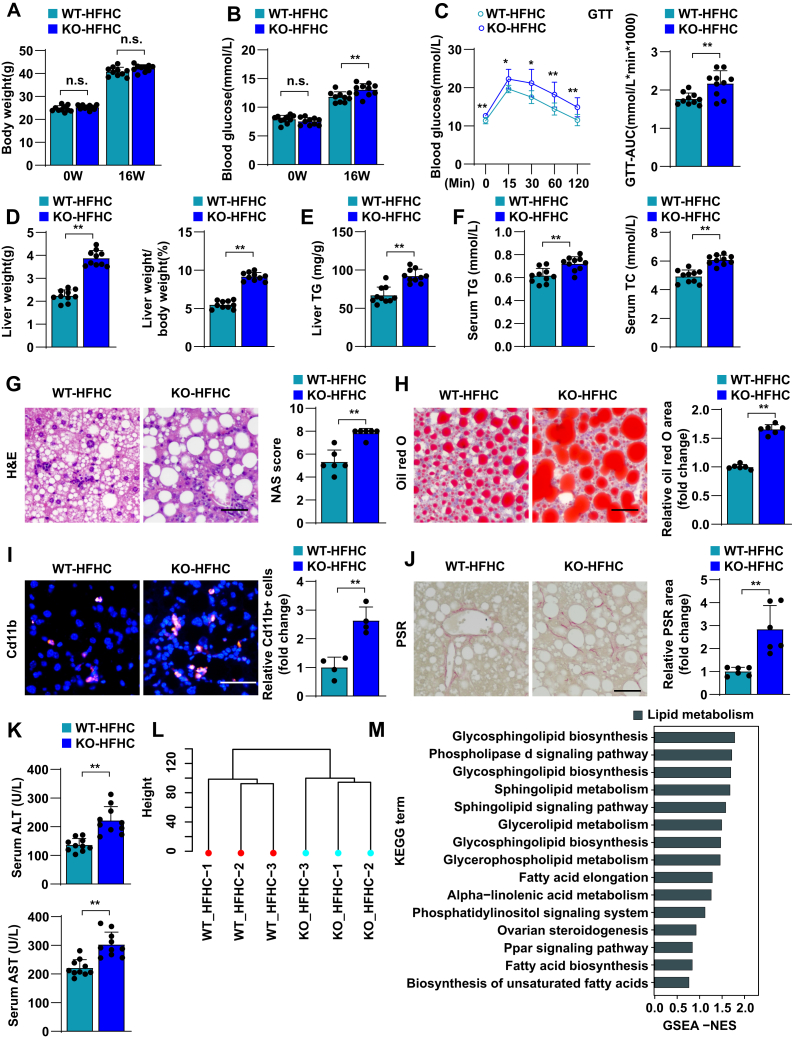

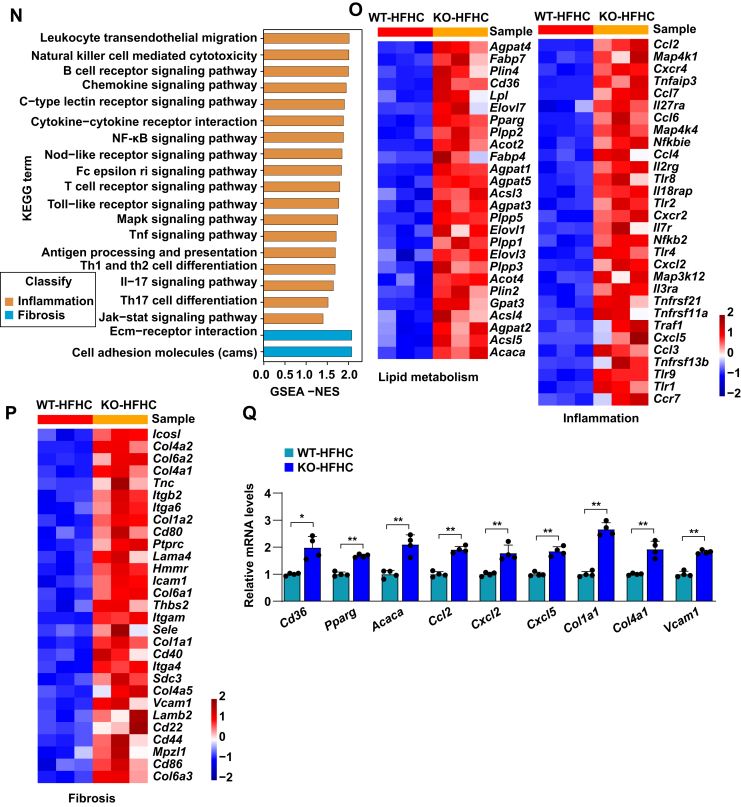
TRIM38 deficiency aggravates HFHC-induced hepatic steatosis, inflammation, and fibrosis. A, B: BWs (A) and fasting blood glucose concentrations (B) of *Trim38*-KO mice and WT control mice after managed with HFHC diet for indicated weeks (*n* = 10 mice/group). C: GTT tolerance was tested in *Trim38*-KO mice and WT control mice at the 15th week of HFHC feeding, and the corresponding areas under the curve were calculated (*n* = 10 mice/group). D: LW (left) and LW-to-BW ratio (right) of *Trim38*-KO mice and WT control mice after fed an HFHC for 16 weeks. *n* = 10 mice/group. E, F: Liver TG (E), serum TG (F—left), and serum TC (F—right) levels in different groups. *n* = 10 mice/group. G, H: Representative images of H&E staining (G) and Oil Red O staining (H) of liver sections of HFHC-fed *Trim38*-KO and WT control mice. NAS scores and Oil Red O area were quantified, respectively. *n* = 6 mice/group. Scale bar represents 50 μm. I: Representative immunofluorescence images of Cd11b (red) in the livers of mice from the indicated groups, nuclei were stained with 4',6-diamidino-2-phenylindole (DAPI) (blue). Cd11b-positive cells per field were quantified, respectively. *n* = 4 mice/group. Scale bar represents 50 μm. J: Representative images of Picrosirius red (PSR) staining in liver sections of HFHC-fed *Trim38*-KO mice and WT mice. *n* = 6 mice/group. Scale bar represents 50 μm. K: The serum levels of ALT and AST in the indicated mice groups. *n* = 10 mice/group. L: Hierarchical clustering showed the distribution and distances of liver samples from *Trim38*-KO mice and WT control mice post 16 weeks feeding of HFHC. M, N: GSEA showed the major activated pathways involved in lipid metabolic processes, inflammatory response, and fibrosis according to the transcriptomics data in liver tissue from indicated mice groups. O, P: Heatmap exhibited the leading genes involved in lipid metabolic pathway, inflammation-related pathway of HFHC-induced KO mice compared to HFHC-induced WT mice from RNA-Seq datasets. Q: Relative mRNA levels of genes related to lipid metabolism, inflammatory response, and fibrosis in the indicated groups. *n* = 4 mice/group. The mRNA expression levels were normalized to those of *ACTB*. The data are presented as the mean ± SD values (∗*P* < 0.05, ∗∗*P* < 0.01, n.s., no significance). The data were statistically analyzed with a two-tailed Student’s test.

In addition to excessive hepatic fat accumulation, NASH is an advanced form of NAFLD along with inflammation and liver cell damage. To further investigate the critical function of TRIM38 in NASH in vivo, a mouse model of HFHC diet-induced NASH was established to exhibit more profound inflammatory responses and fibrosis symptoms. Consistent with our observations in the HFD model, TRIM38 depletion showed no effects on BW after HFD (Fig. 3A). Similarly, *TRIM38*-KO aggravated the HFHC-induced glucose metabolic disorder, as evidenced by increased blood glucose levels (Fig. 3B), AUC in GTT (Fig. 3C). *Trim38*-KO strain also exhibited higher LW and LW/BW (Fig. 3D) and increased TG or TC concentration in the livers and serum (Fig. 3E, F). Moreover, histological staining of H&E, Oil Red O staining, CD11b, and Picrosirius red staining assays demonstrated that TRIM38 depletion exacerbated HFHC diet-induced hepatic steatosis, inflammation, and fibrosis (Fig. 3G–J). *Trim38*-KO also aggravated the HFHC-induced liver injury, as marked by increased serum ALT and AST (Fig. 3K).

RNA-Seq assay analysis also showed a clear separation of the *Trim38*-KO group from the WT group (Fig. 3L) and demonstrated significantly activated lipid metabolism and inflammatory response. Importantly, we noticed that lipid metabolism-related pathways (including glycosphingolipid biosynthesis, phospholipase signaling pathway, glycerolipid metabolism, glycerolipid metabolism, etc.), inflammation, and fibrosis-related pathways (NF-κB signaling pathway, B-cell receptor signaling pathway, IL-17 signaling pathway, Tnf signaling pathway, Mapk signaling pathway, T-cell receptor signaling pathway, Ecm-receptor interaction, cell adhesion molecules pathway, etc.) were simultaneously activated in livers from mice with *Trim38* KO compared with control groups (Fig. 3M, N). Correspondingly, the leading genes of these pathways (Fig. 3O, P) and the representative genes (Fig. 3Q) were significantly activated in the KO-HFHC group. Taken together, these results indicate that TRIM38 deletion exacerbates hepatic steatosis, inflammation, and fibrosis under metabolic stress.

### TRIM38 inhibits lipid accumulation and inflammation in hepatocytes in vitro

**Fig. 4:**
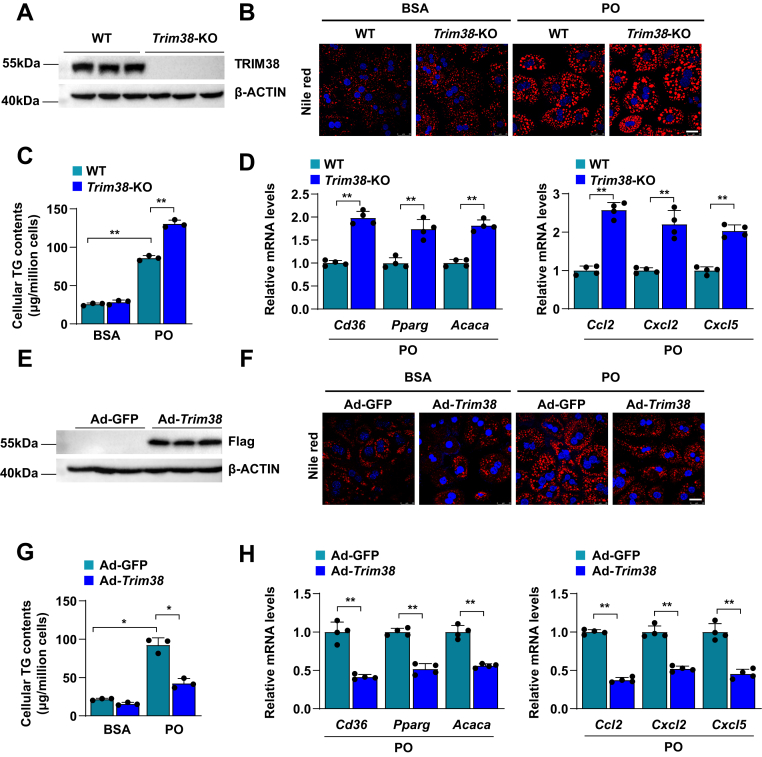
TRIM38 inhibits lipid accumulation and inflammation in hepatocytes in vitro. A: Representative WB analysis for *Trim38*-KO detection in the indicated groups. β-actin was used as control, *n* = 3 independent experiments. B: Representative images of Nile Red staining of control and *Trim38*-KO primary hepatocytes treated with BSA or PO (PA/OA). *n* = 3 independent experiments. Scale bar represents 25 μm. C: The contents of TG in *Trim38*-KO primary hepatocytes and the corresponding control cells (Ctrl) treated with BSA or PO for 12 h. *n* = 3 independent experiments. D: Relative mRNA levels of lipid metabolism genes and inflammatory response genes in *Trim38*-KO primary hepatocytes under PO pressure. *n* = 3 independent experiments. The mRNA expression levels were normalized to those of *ACTB*. E: Representative WB analysis for Trim38 overexpression detection in the indicated groups. β-actin was used as control, *n* = 3 independent experiments. F: Representative images of Nile Red staining of control and TRIM38-overexpression primary hepatocytes treated with BSA or PO. *n* = 3 independent experiments. Scale bar represents 25 μm. G: The contents of TG in TRIM38-overexpression primary hepatocytes and the corresponding control cells (Ctrl) treated with BSA or PO for 12 h. *n* = 3 independent experiments. H: Relative mRNA levels of lipid metabolism genes and inflammatory response genes in TRIM38-overexpression primary hepatocytes under PO pressure. *n* = 3 independent experiments. The mRNA expression levels were normalized to those of *ACTB*. The data are presented as the mean ± SD values (∗*P* < 0.05, ∗∗*P* < 0.01, n.s., no significance). Statistical analysis was performed with a two-tailed Student’s test.

To confirm the observations from RNA-Seq analysis that TRIM38 was involved in the process of lipid metabolism and inflammatory response, we analyzed the effects of TRIM38 on lipid accumulation and inflammation in primary hepatocytes from *Trim38*-KO mice or primary hepatocyte overexpression of TRIM38 by adenovirus infection. The depletion of TRIM38 was confirmed by WB (Fig. 4A). PA/OA stimulation was used to establish cell models, and then the Nile red staining was performed, and results showed that TRIM38 ablation promoted PA/OA-induced lipid accumulation in hepatocytes (Fig. 4B), and the cellular TG content measurement showed higher levels compared with the BSA-treated control group (Fig. 4C). Consistently, qRT-PCR revealed the mRNA level of lipid metabolism-related genes (*Pparg*, *Acaca*, and *Cd36*), and inflammation-related genes (*Ccl2*, *Cxcl2*, and *Cxcl5*) were upregulated in *Trim38*-KO hepatocytes subjected to PA/OA treatment (Fig. 4D). Inversely, we overexpressed TRIM38 in primary hepatocytes by adenovirus infection and confirmed the protein level of Trim38 by WB (Fig. 4E). TRIM38 overexpression affected hepatocytes in the opposite manner, as evidenced by the reduced lipid accumulation and cellular TG level (Fig. 4F, G). The mRNA level of lipid metabolism and inflammation-related gene expression was also consistently repressed by TRIM38 overexpression (Fig. 4H). Taken together, these findings suggest that TRIM38 might inhibit lipid accumulation and inflammation in hepatocytes.

### TRIM38 negatively regulates MAPK signaling pathway by interacting with TAB2 in NASH

**Fig. 5:**
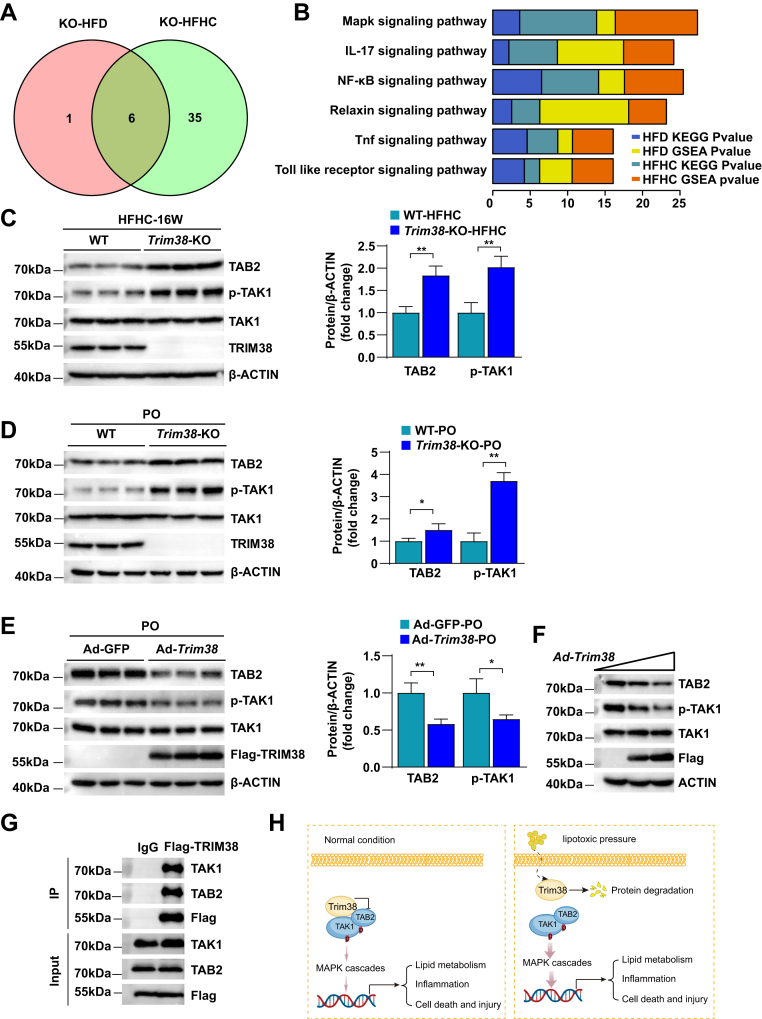
TRIM38 negatively regulates MAPK signaling pathway by interacting with TAB2 in NASH. A: Venn diagram showing the intersection of the six representative KEGG pathways identified by analysis of the transcriptome data in *Trim38*-KO mice fed with HFD for 24 weeks and HFHC diet for 16 weeks based on the RNA-Seq dataset. B: KEGG and GSEA pathway enrichment analysis results (top six enriched pathways) of the transcriptome data. C: Representative WB analysis (left) and quantification (right) of total and phosphorylated TAK1 and TAB2 in liver of WT and *Trim38*-KO mice fed with HFHC for 16 weeks, β-actin was used as control, *n* = 3 mice per group. D, E: WB analysis (left) and quantification (right) of total and phosphorylated TAK1 and TAB2 in PO (PA/OA)-stimulated *Trim38*-KO (D) and *Trim38*-overexpressed (E) primary hepatocytes, β-actin was used as control, *n* = 3 independent experiments. F: The effect of *Trim38* overexpression on TAB2-TAK1 signaling activation in mouse primary hepatocytes was assayed by WB analysis, *n* = 3 independent experiments. G: Semiendogenous IP assays were performed to evaluate the binding of TRIM38 and TAB2 in primary mouse hepatocytes transfected with FLAG-TRIM38 under PA-induced metabolic stress, *n* = 3 independent experiments. H: A summary diagram to illustrate the specific mechanism by which TRIM38 regulates TAB2-TAK1-MAPK pathway. The data are presented as the mean ± SD values (∗*P* < 0.05, ∗∗*P* < 0.01, n.s., no significance). Statistical analysis was performed with a two-tailed Student’s test.

Based on the important role of TRIM38 in NAFLD, we systematically analyzed the data of RNA-Seq datasets of *Trim38*-KO mice. GSEA and KEGG pathway enrichment analysis was performed to reveal the significant changed pathways in the livers of both HFD-fed and HFHC-fed KO mice. The Venn diagram clearly showed that six common pathways were enriched in these two datasets. Among them, MAPK signaling pathway was the most significantly enriched among the top pathways, which was activated through phosphorylation modification (Fig. 5A, B). To further investigate the molecular regulatory mechanism by which TRIM38 affects its downstream pathway, we examined the activation of MAPK-related molecules in HFHC-induced animal models and PA/OA-stimulated cell models. Previous reports indicated that TAB2, a critical adaptor in the activation of TAK1-MAPK cascade, was downregulated by TRIM38 in innate immunity (15). We thus detected the activity of TAB2-TAK1 pathway in the liver samples of HFHC-fed *Trim38*-KO and WT mice. As shown in Fig. 5C, TRIM38 depletion increased the protein abundance of TAB2 and promoted the activation of TAK1. Similar conclusions were obtained in Trim38 depletion and overexpression of hepatocytes (Fig. 5D, E). In addition, TRIM38 overexpression dose-dependently downregulated the protein level of TAB2 and inhibited the phosphorylation of TAK1 (Fig. 5F). Further IP assays were carried out to validate the interaction of the candidate proteins with TRIM38 and revealed that TRIM38 strongly interacted with TAB2 and TAK1 (Fig. 5G). A summary diagram was displayed to illustrate the specific mechanism by which TRIM38 regulates TAB2-TAK1-MAPK pathway (Fig. 5H). Collectively, these data suggest TAB2 as a new target for TRIM38 in NASH and indicate that TRIM38 can interact with TAB2 and promoted its protein degradation, thus attenuating the activation of the MAPK signaling pathway. These results are consistent with the hypothesis that TRIM38 promotes degradation of TAB2 to impact lipid accumulation in models of high-fat-induced steatosis.

### TAB2 abrogates the inhibitory effects on lipid accumulation and inflammation in TRIM38 overexpression hepatocytes

**Fig. 6:**
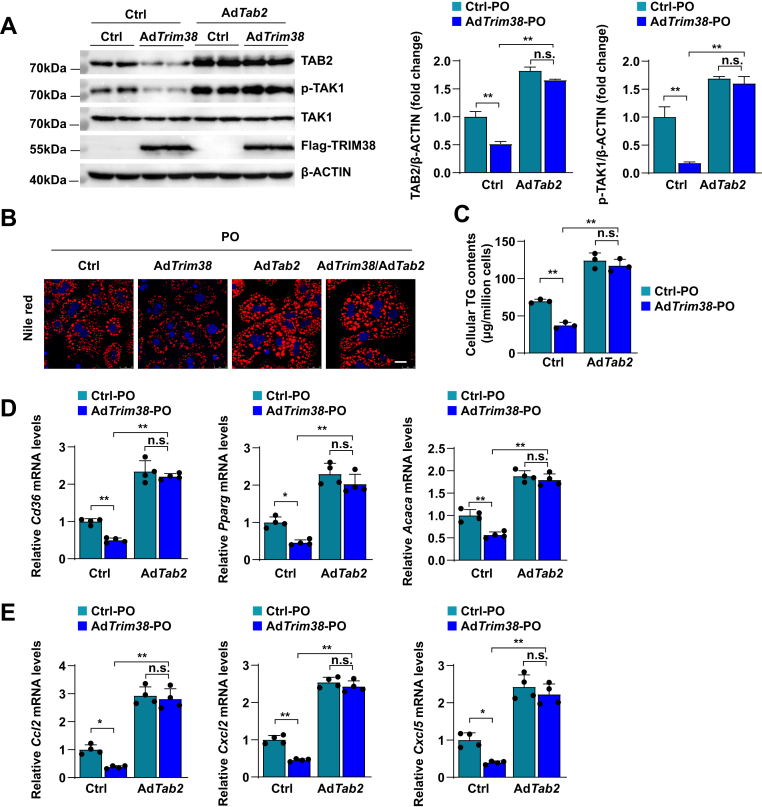
TAB2 overexpression attenuates the protective roles of TRIM38 on NAFLD. A: Representative WB analysis (left) and quantification (right) of total and phosphorylated TAK1 and total TAB2 in primary hepatocytes infected with adenovirus of Ad-*Tab2* and Ad-*Trim38* after PO (PA/OA) stimulation, *n* = 3 independent experiments. B: Representative images of Nile Red staining of primary hepatocytes in indicated group treatment with PO. *n* = 3 independent experiments. Scale bar represents 25 μm. C: The contents of TG in primary hepatocytes of corresponding groups induced by PO for 12 h. *n* = 3 independent experiments. D-E: Relative mRNA levels of lipid metabolism genes and inflammatory response genes in primary hepatocytes of corresponding groups under PO pressure. *n* = 3 independent experiments. The mRNA expression levels were normalized to those of *ACTB*. The data are presented as the mean ± SD values (∗*P* < 0.05, ∗∗*P* < 0.01, n.s., no significance). Statistical analysis was performed with a two-tailed Student’s test.

Since we proved that TRIM38 directly interacts with TAB2 and associates with its degradation, we overexpressed Tab2 via adenovirus infection in Trim38-overexpressed primary hepatocytes. WB analysis demonstrated that TAB2 rescued the TRIM38-mediated inhibitory effects on TAK1 phosphorylation (Fig. 6A). Further Nile red staining showed that overexpression of TAB2 reversed the inhibitory effects of TRIM38 on lipid accumulation in PA/OA-stimulated hepatocytes (Fig. 6B). Consistently, compared with the control cells, TAB2 also reversed the lower cellular TG contents in Ad*Trim38* cells (Fig. 6C). Furthermore, TAB2 overexpression markedly reversed the inhibitory of lipid metabolism-related genes (*Pparg*, *Acaca*, and *Cd36*) and inflammation-related genes (*Ccl2*, *Cxcl2*, and *Cxcl5*) expression in Trim38-overexpressed hepatocyte (Fig. 6D, E). Collectively, our results suggested that the protective roles of TRIM38 on NAFLD might depend on the direct target TAB2.

## Discussion

NAFLD is the most prevalent liver disorder in the world and is associated with a high risk of occurrence of other metabolic syndromes, end-stage liver diseases, and extrahepatic cardiovascular diseases (25, 26, 27). As the terminal stage of NAFLD, NASH is recognized as a component of metabolic syndrome, in which metabolic perturbations occurred in the fatty liver, producing systemic metabolic derangement (28). In the present study, we provide the first evidence that TRIM38 is a robust and protective regulator of lipid accumulation in hepatocytes in vitro and in mouse livers in vivo. The vicious cycle resulting from hepatocyte steatosis, insulin resistance, the inflammatory response, and liver fibrosis was aggravated by TRIM38 deficiency in hepatocytes. We further clarified that TRIM38 could directly bind to TAB2 and promote its protein degradation. Therefore, this study clearly indicates that TRIM38 is a novel suppressor in NAFLD progression and targeting TRIM38 represents a promising therapeutic strategy for NAFLD.

Ubiquitin conjugation represents a novel pattern of post-transcriptional modification and is involved in regulation of different cellular processes in addition to proteolytic degradation (29). TRIM family proteins are E3 ubiquitin ligases featuring conserved really interesting-new-gene, B-BOX, and coiled-coiled structures (30), which implicated in a broad range of cellular processes, including cell cycle regulation, transcriptional regulation, protein quality control, immune defense, and inflammatory responses (8, 30). As a typical member of the TRIM protein family, the mechanism of action of TRIM38 has been explored in diverse fields (9). TRIM38 has been shown to be capable of activating NF-κB signaling pathways (31) and may participate in regulating innate immune response, various pathophysiological processes, such as bone proliferation, tumorigenesis, and autoimmune diseases (10, 13).

Hu *et al.* (15) found that TRIM38 negatively regulates TNF-α, and IL-1β triggered MAPK activation by mediating lysosomal-dependent degradation of TGF-β activated kinase 1 (TAK1)-binding protein TAB2/3, two critical components of the TAK1 kinase complex. Thus, MAPK activation may be a key TRIM38 downstream signaling pathway. Abundance evidences demonstrate that TAK1 is a vital upstream activator of the MAPK signaling pathway and participates in the development of NAFLD, and its activation was fine-tuned by ubiquitination (19, 32, 33). Through the combination analysis of the RNA-Seq datasets, we found that MAPK was the predominant downstream pathway of TRIM38 in NAFLD. Based on the previous reports, we first testified the TAB2-TAK1-MAPK cascades in the liver of *Trim38*-KO mice and in vitro hepatocyte samples. Our results revealed that TRIM38 deficiency enhanced TAK1 activity, whereas its overexpression exerted the opposite effects. Therefore, we conclude that the effects of TRIM38 may occur through the inhibition of the TAK1/MAPK axis. It was reported that TAB2 may function as substrate adaptor and directly bind to TRAF6 and TAK1, leading to autophosphorylation and activation of TAK1, and further activation of NF-κB and MAPK pathway (34, 35). TAB2 can recognize the K63-linked ubiquitin chain of RIP1 in the TNF-α signal or TRAF6 in the IL-1β signal and recruit TAK1 to the signalosome (36). Previous research by Hu *et al.* (15) confirmed that TRIM38 promotes the degradation of TAB2 and TAB3, necessary for ubiquitin ligase activity, to prevent the persistent activation of TAK1. Here, we examined whether TRIM38 interacts with TAK1 and TAB2. IP experiments suggested that TRIM38 interacted with TAK1-TAB2 complex and catalyzed the degradation of TAB2, thus negatively regulating the activation of TAK1-MAPK signaling pathway. Furthermore, TAB2-potentiated phosphorylation of TAK1 was attenuated after TRIM38 overexpression, indicating that TRIM38 inhibited TAB2-mediated phosphorylation of TAK1. Previous studies have reported that TRIM38 degrades TAB2 through lysosomal ubiquitination (15), which may also be the possible mechanism of TRIM38 in NAFLD. We propose that TRIM38 catalyzes its targets through ubiquitylation in the cytoplasm, thus regulating signaling pathways or TAB2 complex activation. The precise role and mechanism of TRIM38 in ubiquitylation need to be further addressed.

To further testify whether the effects of TRIM38 in NAFLD depends on TAB2, we overexpressed *Tab2* via adenovirus infection in *Trim38* overexpressed primary hepatocytes. Our results suggested that the protective roles of TRIM38 on NAFLD might depend on the direct target TAB2. Actually further experiments need to be performed in vivo to validate the mechanism of TRIM38-TAB2 axis, and the best verification method to explore whether *Tab2* KO can reverse the function of Trim38 KO is construction of the gene double-KO mice (knocking out *Tab2* gene in *Trim38*-KO mice). However, generating double-KO mice has high technical barriers and takes a long period. In our research, the best novelty in our job is the new function of TRIM38 in the progression of NAFLD and its potential regulatory mechanism. Our future studies will be devoted to in vivo models, which would be a valuable addition to the field and could provide important insights into the role of TRIM38 in NAFLD pathogenesis. Collectively, our results indicate that TRIM38 is a novel regulator of NASH and that its function is mainly to promote the degradation of TAB2 and inhibit the TAK1-MAPK signaling pathway.

However, there is limited research on the upstream regulation of TRIM38. In our study, we first evaluated the expression of TRIM38 on protein levels in variety models of NAFLD–NASH and found that TRIM38 was downregulated in NAFLD–NASH. To further investigate the regulation of TRIM38 expression in response to high-fat or high-fat high-cholesterol diets, we performed additional experiments via qPCR assay to detect the mRNA levels of *Trim38*. Data showed that the mRNA level of *Trim38* was not affected by lipotoxic pressure, suggesting that TRIM38 expression is regulated by other mechanisms, such as post-translational modifications. Previous report indicates that TRIM38 has E3 ubiquitin ligase activity and can be degraded during virus infection, TRIM38 functions as an E3 enzyme and can be self-ubiquitinated (37). This suggests that TRIM38 may also be self-ubiquitinated and degraded in response to lipotoxic pressure during NAFLD progression. Further investigations are needed to elucidate the underlying mechanisms on the upstream regulation of TRIM38, which could be an interesting avenue for our future research.

In conclusion, this study reveals that TRIM38 facilitates the protein degradation of TAB2, which subsequently inhibits the activated TAK1-MAPK cascades in NASH progression. Our study identifies TRIM38 as a novel suppressor of TAB2-TAK1-MAPK signal cascades, which reveals great significance for clinical translation.

## Data availability

The authors declare that the data supporting the findings of this study are available within the article and its supplemental data files.

## Supplemental data

This article contains supplemental data.

## Conflict of interest

The authors declare that they have no conflicts of interest with the contents of this article.
