# Supplementary material for: Tripartite motif 38 alleviates the pathological process of NAFLD–NASH by promoting TAB2 degradation
Source: J Lipid Res. 2023 Apr 26;64(7):100382. doi: 10.1016/j.jlr.2023.100382 (PMC10394331; doi:10.1016/j.jlr.2023.100382)
Supplement: Supplemental Tables S1–S3 [file mmc1.pdf]

Tripartite motif 38 alleviates the pathological process of NASH by promoting TAB2 degradation

Xinxin Yao[1,2#], Ruixiang Dong[1,2,#], Sha Hu[1,2], Zhen Liu[2], Jie Cui[1], Fengjiao Hu[2,3], Xu Cheng[4,5], Xiaoming Wang[1,2], Tengfei Ma[6], Song Tian[1,2], Xiao-Jing Zhang[1,2], Yufeng Hu[4,5], Lan Bai[4,5,*], Hongliang Li[1-4,7,*], Peng Zhang[1,2,*]

[1]Taikang Medical School (School of Basic Medical Sciences), Wuhan University, Wuhan, China;

[2]Institute of Model Animal, Wuhan University, Wuhan, China;

[3]Medical Science Research Center, Zhongnan Hospital of Wuhan University, Wuhan, China;

[4]Gannan Innovation and Translational Medicine Research Institute, Ganzhou, China;

[5]Key Laboratory of Cardiovascular Disease Prevention and Control, Ministry of Education, First Affiliated Hospital of Gannan Medical University, Gannan Medical University, Ganzhou, China;

[6]Department of Neurology, Huanggang Central Hospital, Huanggang, China;

[7]Department of Cardiology, Renmin Hospital of Wuhan University, Wuhan, China;

[#]These authors contributed equally to this work.

***CORRESPONDENCE:**

Peng Zhang, Ph.D.

Taikang Medical School (School of Basic Medical Science), Wuhan University

E-mail: zhp@whu.edu.cn

Hongliang Li, M.D., Ph.D.

Department of Cardiology, Renmin Hospital of Wuhan University

E-mail: lihl@whu.edu.cn

Lan Bai, Ph.D

Gannan Innovation and Translational Medicine Research Institute

E-mail: bailan@gmu.edu.cn

Supplementary Table 1. Antibodies for Western blot analysis

Antibody	Cat No.	Manufacturer
TRIM38	A2600 (1:1000)	ABclonal
β-ACTIN	AC026 (1:50000)	ABclonal
p-TAK1	4508S (1:1000)	CST
TAK1	AB109526 (1:1000)	Abcam
TAB2	A9867 (1:1000)	ABclonal
Flag	M185-3LL (1:1000)	MBL

Supplementary Table2. Primers for qPCR

Gene(mouse)	Primer	Sequence
Trim38	F	CACAGCCCACCTTGACCTAG
	R	CCCAGGACACAGGGTAAACC
Cd36	F	GACTGGGACCATTGGTGATGA
	R	AAGGCCATCTCTACCATGCC
Fabp1	F	TGGTCCGCAATGAGTTCACCCT
	R	CCAGCTTGACGACTGCCTTGACTT
Gpat3	F	TCCTGCTACCTCTGAGGGTC
	R	GTCAGATGCACCAGCTCACT
Pparg	F	ATTCTGGCCCACCAACTTCGG
	R	TGGAAGCCTGATGCTTTATCCCCA
Acaca	F	GGCCAGTGCTATGCTGAGAT
	R	AGGGTCAAGTGCTGCTCCA
Ccl2	F	TACAAGAGGATCACCAGCAGC
	R	ACCTTAGGGCAGATGCAGTT
Cxcl2	F	GCGCCCAGACAGAAGTCATA
	R	CAGTTAGCCTTGCCTTTGTTCA
Cxcl5	F	CGGTTCCATCTCGCCATTCA
	R	GCTATGACTGAGGAAGGGGC
Col1a1	F	TGCTAACGTGGTTCGTGACCGT
	R	ACATCTTGAGGTCGCGGCATGT
Col4a1	F	AACAACGTCTGCAACTTCGC
	R	CTTCACAAACCGCACACCTG

Vcam1	F	ATTTTCTGGGGCAGGAAGTT
	R	ACGTCAGAACAACCGAATCC

Supplementary Table3. Primers for adenovirus construction

Gene(mouse)	Primer	Sequence
Ad-*Trim38*	F	GGCTAGCGATATCGGATCCGCCACCATGGGCTCAGACTTTAGCACGG
	R	CGTCCTTGTAATCACTAGTTTGATTATTTATGGCAGGCAGGAAC
Ad-*Tab2*	F	GCTAGCGATATCGGATCCGCCACCATGGCCCAAGGAAGCCAC
	R	ACTAGTGGTACCAAGCTTGAAATGCCGAGGCATCTCACAC
